# The use of S_2_O_8_^2−^ and H_2_O_2_ as novel specific masking agents for highly selective “turn-on” fluorescent switching recognition of CN^−^ and I^−^ based on Hg^2+^–graphene quantum dots

**DOI:** 10.1039/c7ra12327b

**Published:** 2018-01-03

**Authors:** Prawit Nuengmatcha, Phitchan Sricharoen, Nunticha Limchoowong, Ratana Mahachai, Saksit Chanthai

**Affiliations:** Materials Chemistry Research Center, Department of Chemistry and Center of Excellence for Innovation in Chemistry, Faculty of Science, Khon Kaen University Khon Kaen 40002 Thailand sakcha2@kku.ac.th +66-4320-2373 +66-4300-9700 ext. 42174-5

## Abstract

In this study, we report that both CN^−^ and I^−^ can enhance the fluorescent intensity of Hg^2+^–graphene quantum dots (Hg^2+^–GQDs). However, the selectivity of the sensor was poor. Accordingly, simple specific masking agents can be directly used to solve this problem. Here, for the first time, we report the use of persulfate ion (S_2_O_8_^2−^) as a turn-on fluorescent probe of Hg^2+^–GQDs for selective CN^−^ detection, while hydrogen peroxide (H_2_O_2_) was selected for its sensing ability towards I^−^ ion detection. Interestingly, the signal was immediately measured after addition of the masking agent to Hg^2+^–GQDs and the sample because its interaction was very fast and efficient. The method had a linear response in the concentration ranges of 0.5–8 μM (*R*^2^ = 0.9994) and 1–12 μM (*R*^2^ = 0.9998) with detection limits of 0.17 and 0.20 μM for CN^−^ and I^−^, respectively. The sensor was successfully used for the dual detection of both CN^−^ and I^−^ in real water samples with satisfactory results. In conclusion, the specific masking agents in a Hg^2+^–GQDs system appeared to be good candidates for fluorometric “turn-on” sensors for CN^−^ and I^−^ with excellent selectivity over other ions.

## Abstract 2

In this study, we report that both CN^−^ and I^−^ can enhance the fluorescent intensity of Hg^2+^–graphene quantum dots (Hg^2+^–GQDs).

## Graphical abstract

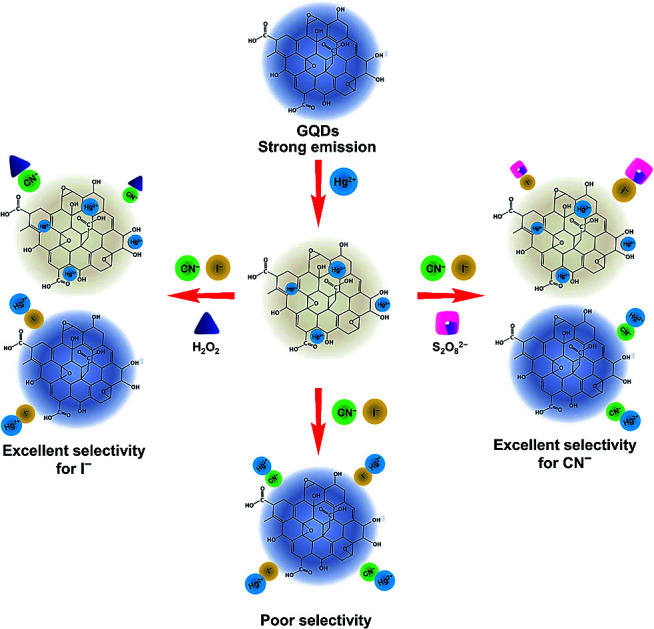

## Introduction

Graphene quantum dots (GQDs) are single atom-thick graphene sheets with a size of less than 10 nm, similar to other graphene nanosheets.^1^ They are the most commonly used optical sensing nanomaterials because of their high extinction coefficients and various alluring properties, such as high surface area, electrical conductivity, thermal conductivity and photostability, excellent chemical stability, environmental friendliness and good biocompatibility.^2,3^ Moreover, their surface is versatile and can be easily immobilized with both various organic functional groups and metal nanomaterials, including glucose oxidase,^4^ hyaluronic acid,^5^ amino group,^6,7^ carboxylic group and nitrite,^8^ polyaniline,^9^ gold nanoparticles,^10^ platinum nanoparticles^11^ and silver nanoparticles^12^ through strong covalent bonding or physical adsorption. As a result, many GQDs-based fluorescent sensors have been developed to detect metal ions (*e.g.* chromium(vi),^1^ mercury(ii),^13^ copper(ii),^14^ iron(iii),^15^) biomolecules (*e.g.* dopamine,^16^ microcystin-LR,^17^ DNA,^18^ doxorubicin^19^ and biothiols^20^) and other analytes (*e.g.* phenol,^21^ trinitrophenol^22^ and uric acid^23^). However, the application of GQDs usually involves tedious processes for the dual detection of target analytes. Thus, the dual detection of GQDs sensors is needed urgently for the trace analysis of cations, anions, molecules and biomacromolecules.

The selective sensing of anions, such as fluoride (F^−^), chloride (Cl^−^), bromide (Br^−^), iodide (I^−^), acetate (AcO^−^) and cyanide (CN^−^) ions, is highly important because they are widely distributed and play important roles in biological, environmental and chemical industries.^24–26^ In particular, iodide is an important microelement to humans, as it plays a key role in several biological activities, such as brain function, muscle tissue growth, neurological activity and thyroid function.^27^ In addition, iodide helps to maintain the release of the thyroid hormone into the bloodstream. Either deficiency or excess of iodine intake would cause major health problems. For example, iodine deficiency in pregnancy will cause spontaneous abortion, fetal goitre, cretinism, anxiety and nervous agitation, intellectual impairment and neonatal hypothyroidism, while iodine excess will lead to hyperthyroidism. These disorders can be prevented by ensuring optimal iodide intake.^28,29^ With regard to cyanide, it is the most threatening to the environment and human life. Various products are very high in cyanide due to their industrial uses, including the production of paper, textiles, plastics and nitriles, metals, electroplating and the extraction of gold and silver. In addition, cyanide is also released from biological processes of bacteria, fungi.^30^ Due to cyanide being an extremely lethal poison to humans and aquatic life, the World Health Organization (WHO) permits the maximum acceptable concentration level (0.2 ppm) of cyanide in drinking water^30^ as does the U.S. Environmental Protection Agency (EPA), which regulates an ultra-trace level (5 ppb) of cyanide in the environmental primary standards.^31^

To date, many strategies have been proposed for the detection of I^−^ and CN^−^ in aqueous samples, such as ion chromatography,^32^ liquid chromatography-mass spectrometry,^33^ high resolution nuclear magnetic resonance spectroscopy and liquid chromatography,^34^ indirect atomic absorption spectrometry,^35^ Raman scattering,^36^ UV-vis spectrophotometry,^37^ gas chromatography,^38^ electrospray ionization tandem mass spectrometry,^39^ flow injection-flame atomic absorption spectrometry,^40^ inductively coupled plasma-optical emission spectrometry^41^ and adsorptive stripping voltammetry.^42^ However, these techniques are rather time-consuming and require tedious sample preparation and specific operating skills. Moreover, the performance of some techniques is seriously affected by interferences of coexisting anions, while more complex and expensive instruments are mandatory for others.

Recently, the fluorescent sensor has been shown to be a promising technique and has been widely applied for cyanide and iodide detection. For example, a new fluorescent cyanide chemosensor based on a phenothiazine derivative,^43^ a turn-on fluorescent sensor for cyanide detection based on BODIPY-salicylaldehyde,^44^ a red-emitting fluorescent sensor for cyanide based on a hybrid naphthopyran–benzothiazol,^45^ a turn-on fluorescent probe for cyanide based on the aggregation of terthienyl,^46^ fluorescence turn-on chemosensor for cyanide based on pyridine cation,^47^ iodide determination using a novel LSPR fluorescent Ag nanocluster probe,^48^ ultrasensitive fluorescent detection of iodide using conjugated polyelectrolyte-stabilized silver nanoparticles coupled with pyrene derivative^49^ and a turn-on fluorescent sensor for iodide detection based on newly synthesized oligopyrrole derivative.^50^ Nevertheless, these reports show suitable processes for the detection of only single cyanide or iodide. Consequently, the dual detection of a fluorescent sensor is not only in high demand but also remains a challenge for the trace analysis of both CN^−^ and I^−^ in real samples.

This study was aimed towards developing Hg^2+^–graphene quantum dots (Hg^2+^–GQDs) as a fluorescent turn-on sensor for the selective detection of both I^−^ and CN^−^ ions. Normally, GQDs give strong blue photoluminescence in an aqueous solution. It was also observed that Hg^2+^ could drastically quench the fluorescent intensity of GQDs. When both I^−^ and CN^−^ were added to an assay solution, they could selectively interact with Hg^2+^, resulting in that the Hg^2+^–GQDs complex might be destroyed and the turn-on fluorescent intensity recovered. The fluorescent intensity of the GQDs was linearly related to their concentration ranges of both I^−^ and CN^−^. However, in the presence of specific masking reagents, such as persulfate (S_2_O_8_^2−^) or hydrogen peroxide (H_2_O_2_), the GODs sensor was selectively capable of detecting trace levels of only I^−^ or CN^−^, respectively. The practical feasibility of this approach was also demonstrated for the analysis of I^−^ and CN^−^ in drinking water samples.

## Experimental

### Chemicals

Citric acid, sodium hydroxide, silver chloride, potassium chloride and barium chloride were purchased from Ajax Fine Chem Pty. Ltd. Mercury nitrate, cobalt nitrate hexahydrate, lead nitrate, zinc nitrate hexahydrate, cadmium nitrate tetrahydrate, copper nitrate trihydrate, potassium cyanide and potassium iodide were purchased from Sigma-Aldrich and used without further purification.

### Instrumentation

UV-visible absorption spectra were obtained with an Agilent 8453 spectrophotometer (Agilent, Germany). Emission spectra were recorded using a RF-5301PC spectrofluorophotometer (Shimadzu, Japan), with an excitation wavelength of 370 nm. The excitation and emission slit widths were 5 nm. A quartz cuvette with a 1 cm path length and 1 cm window width was used for the UV-visible and fluorescence measurements. Transmission electron microscopy (TEM) images were obtained using a JEOL 1200 electron microscope operating at an accelerating voltage of 200 kV (JEOL Ltd., Japan). The functional groups of GQDs were characterized by attenuated total reflectance-Fourier transform infrared (ATR-FTIR) spectroscopic measurements using a TENSOR 27 system Fourier transform infrared spectrometer (Bruker, Germany).

### Preparation of GQDs

GQDs were prepared from citric acid by a pyrolysis method with a modified procedure.^20^ Briefly, 2.0 g of citric acid was added into a 5 mL vial. The vial was heated to 260 °C using a paraffin oil bath for about 10 min. The citric acid was slowly liquated to a yellow colour. The liquid was transferred into a beaker containing 100 mL of 0.25 mol L^−1^ NaOH with continuous stirring for 30 min. The obtained sample solution was neutralized to pH 7.0 with NaOH, and the GQDs stock solution was stored at 4 °C before use.

### Quenching of GQDs using Hg^2+^

First, 20 μL of 2.0 mg mL^−1^ GQDs solution and 1 mL of 1 mol L^−1^ phosphate buffer pH 7.4 were mixed in 10 mL volumetric flask. Then, various concentrations of Hg^2+^ were added to an aliquot of the GQDs solution (10 mL final volume) at room temperature. The Hg^2+^-quenched fluorescent spectrum of each GQDs solution was recorded immediately at *λ*_ex_/*λ*_em_ 370/465 nm. Then, such spectral measurements were used to plot the quenching calibration curve for Hg^2+^.

### Detection of I^−^ and CN^−^

Various concentrations of either I^−^ or CN^−^ and 20 μL of the Hg^2+^-quenched GQDs solution were mixed in 0.1 mol L^−1^ phosphate buffer solution adjusted to various pH values (10 mL final volume). For the selective determination of I^−^ and CN^−^, a specific masking agent was chosen for the anion solution prior to recording the fluorescent spectrum of each solution at *λ*_ex_/*λ*_em_ 370/465 nm. Such spectral measurements were used to plot the enhancing calibration curves for both I^−^ and CN^−^. In addition, the optimum conditions for the dual detection of CN^−^ and I^−^ were also investigated in detail. Moreover, to evaluate the performance of the proposed method, the optimized conditions were validated in terms of linearity, limits of detection and quantification, and precision (expressed as the relative standard deviation, RSD, of the calibration slope obtained from both intra-day and inter-day analysis). Finally, the Hg^2+^–GQDs-based sensor was applied for the analysis of CN^−^ and I^−^ in real water samples.

## Results and discussion

### Characterization of GQDs

**Fig. 1:**
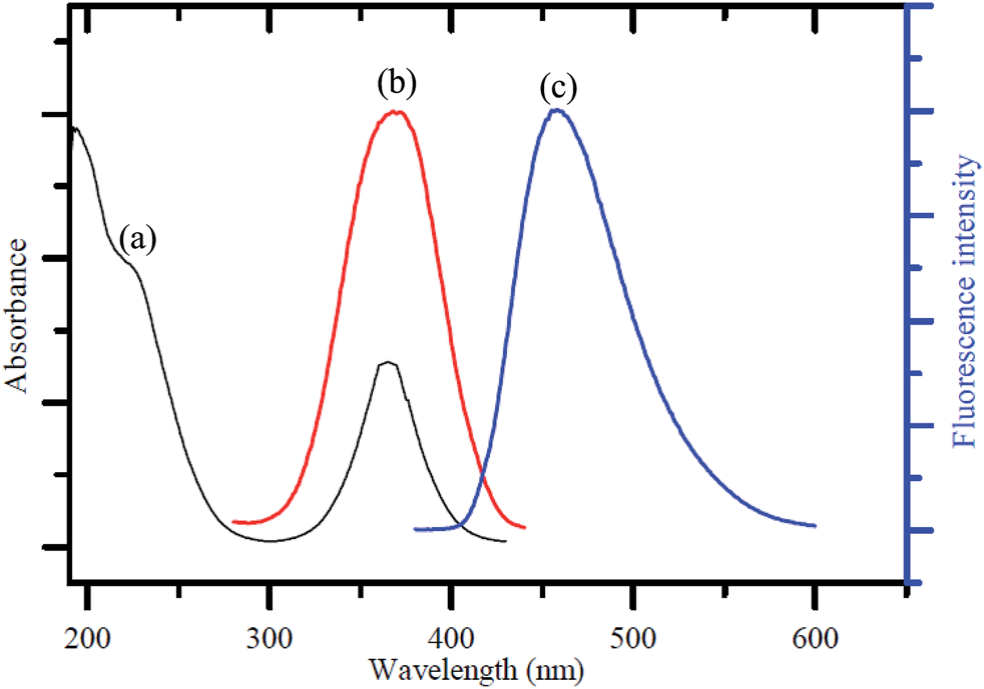
(a) UV-visible absorption spectrum, (b) PL excitation and (c) emission spectra of the GQDs.

Fig. 1a shows the UV-visible absorption spectrum of the as-synthesized GQDs, giving two absorption bands at around 230 nm and 365 nm. A broad band appeared at 230 nm, resulting in nearly no fluorescence signal, which was attributed to π–π* transition of the aromatic sp^2^ domains.^1^ The other typical absorption peak was at 365 nm, which was assigned to the n–π* transition of graphitic sp^2^ domains.^51,52^ The photoluminescence (PL) spectrum of the GQDs showed a strong peak at 465 nm (Fig. 1b) when excited at 370 nm (Fig. 1c), and the full width at half maximum (FWHM) was about 100 nm, which resembles that of most graphene quantum dots.^52–54^

**Fig. 2:**
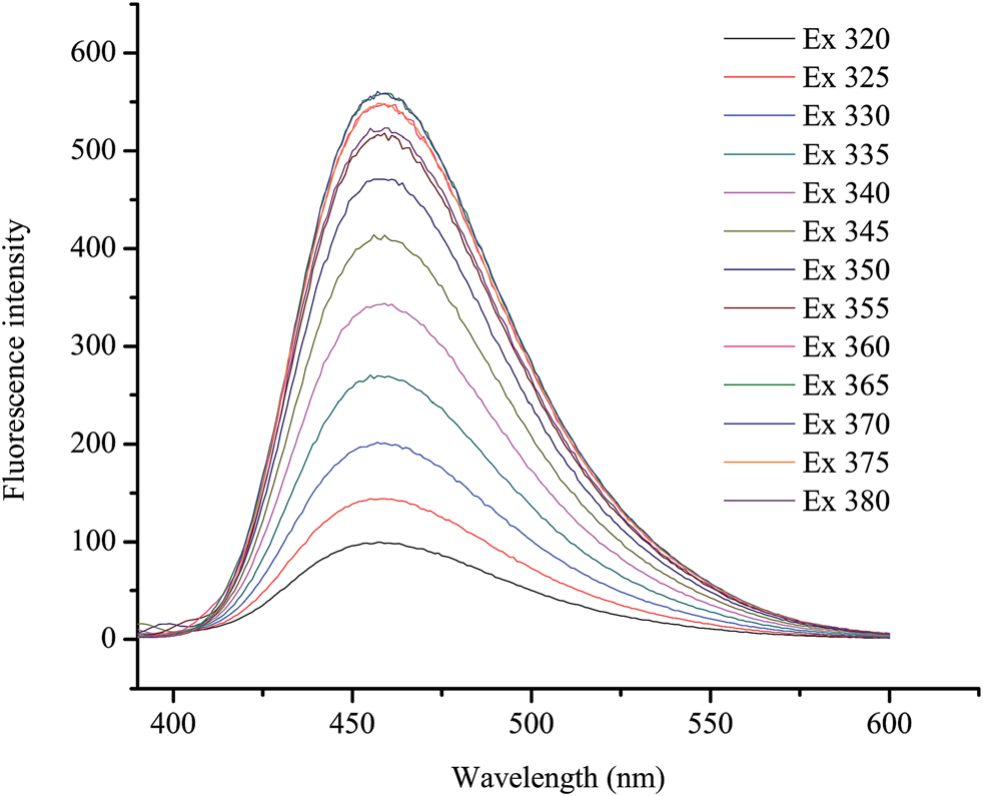
PL emission spectra of the GQDs at different excitation wavelengths.

To further characterize the optical properties of the as-synthesized GQDs, detailed PL investigations were carried out with different excitation wavelengths (Fig. 2). When the excited wavelengths changed from 320 to 380 nm, the PL intensity decreased remarkably, but the fluorescent peak was not shifted. This excitation-independent PL behaviour had a similar trend compared with most of reported GQDs.^55–57^

**Fig. 3:**
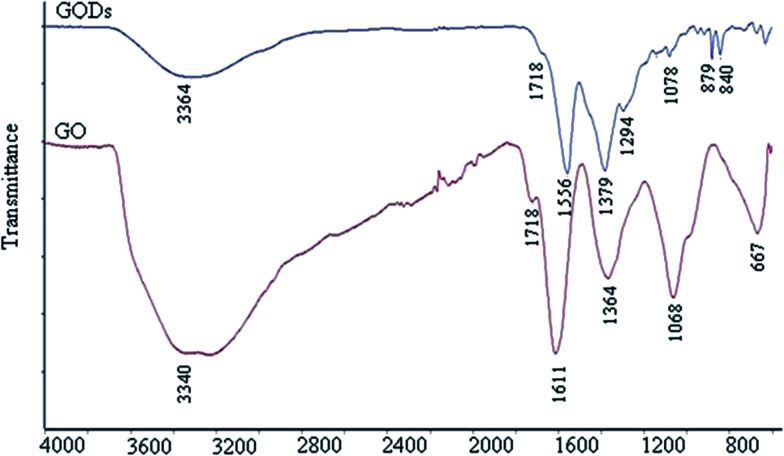
FT-IR spectra of the GQDs and GO.

Fig. 3 shows the FT-IR spectra of the GQDs and graphene oxide (GO) as a reference. From both spectra, there are strong bands at around 1556 cm^−1^ due to the C=C stretching mode of the polycyclic aromatic hydrocarbons,^19,58^ indicating that GQDs remain the structure of graphene. Moreover, the bands at around 1718 cm^−1^ corresponded to the carboxyl group, and the bands at around 3440 cm^−1^ were attributed to the stretching vibration of the hydroxyl group. The peaks at 1364–1068 cm^−1^ were attributed to the C–O in the COH/COC (epoxy) group.^59^ For comparison, the bands of carboxyl and hydroxyl groups for GQDs are dramatically reduced compared with GO but still exist, while those of the epoxy group have almost disappeared. Therefore, it can be speculated that the GQDs exhibit the main sheet structure of graphene, with rare carboxyl and hydroxyl groups remaining on the sheets.^19^

**Fig. 4:**
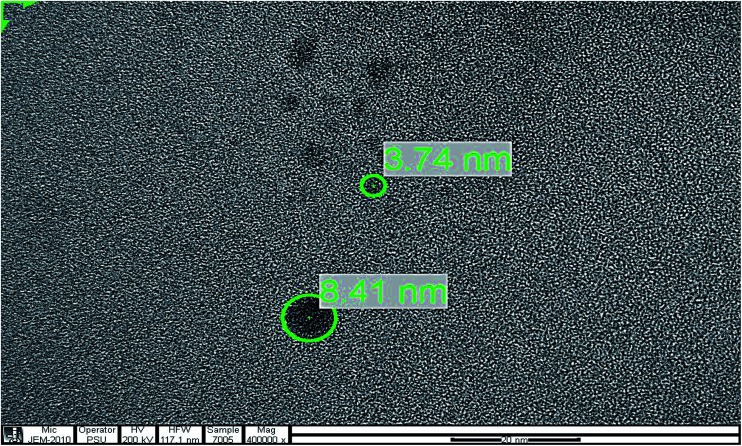
TEM image of the GQDs.

To determine the particle size of the GQDs, the obtained sample was characterized by transmission electron microscopy (TEM). Fig. 4 shows the image of the GQDs. Using image J software, the GQDs particles with diameters in the range of 3.74 nm to 8.41 nm were distributed uniformly. The small particle sizes of GQDs exhibited a characteristic of their fluorescent intensity.

### The quenching effect of various metal ions on the fluorescent intensity of GQDs

**Fig. 5:**
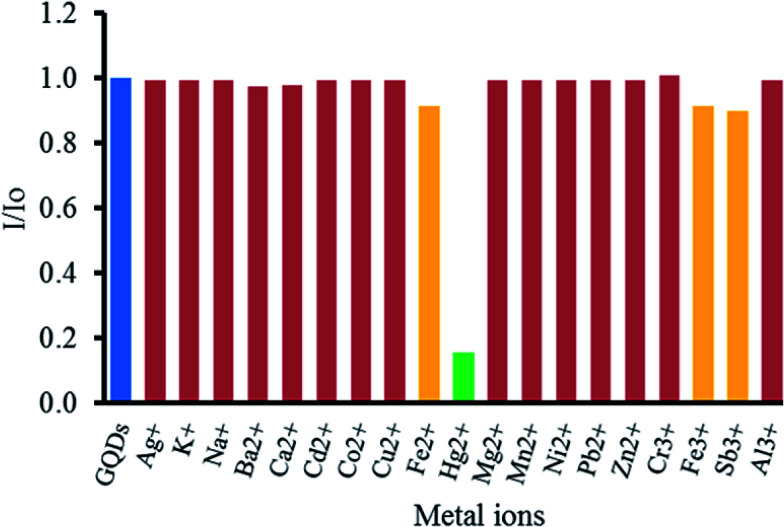
Quenching of the fluorescent intensity of the GQDs by different metal ions. All the ions were at 100 μM concentration. *I* and *I*_0_ are the emission intensities of the GQDs at 465 nm either in the presence or in the absence of some metal ions in 0.1 M phosphate buffer (pH 7.4).

To study the effect of metal ions on the quenching of GQDs, various metal ions, including Ag^+^, K^+^, Na^+^, Ba^2+^, Ca^2+^, Cd^2+^, Co^2+^, Cu^2+^, Fe^2+^, Fe^3+^, Hg^2+^, Mg^2+^, Mn^2+^, Ni^2+^, Pb^2+^, Zn^2+^, Cr^3+^, Fe^3+^, Sb^3+^ and Al^3+^ ions were tested. From Fig. 5, it was found that Hg^2+^ could strongly quench the fluorescent intensity of the GQDs. Both Fe^2+^ and Fe^3+^ had slight quenching effects. Other metal ions gave rather a low one. That high selectivity may be ascribed to the fact that Hg^2+^ possesses a stronger affinity towards the carboxyl groups on the GQDs surface than other metal ions,^60^ and the selective quenching presumably occurs *via* either electron or energy transfer from GQDs to Hg^2+^.^61^

### Optimization conditions for the quenching effect of Hg^2+^ on the GQDs fluorescent sensor

**Fig. 6:**
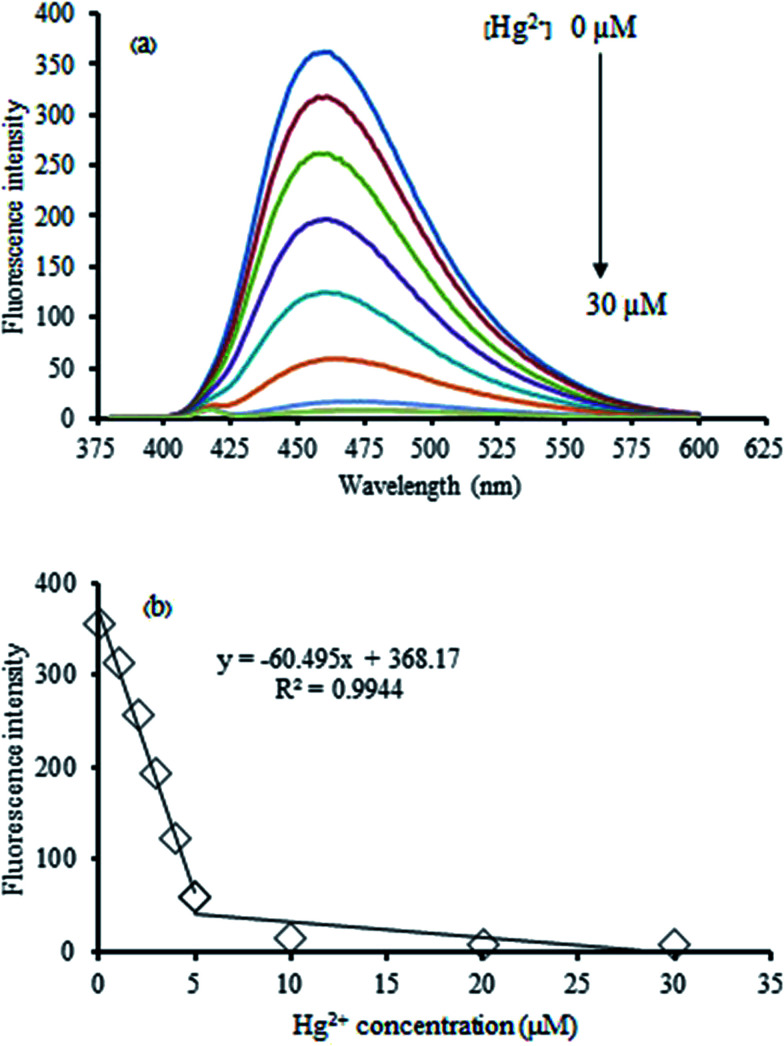
(a) Changes in the emission spectra of the GQDs at different Hg^2+^ concentrations (0–30 μM), and (b) changes in the emission intensity at 465 nm *versus* Hg^2+^ concentration in 0.1 M phosphate buffer (pH 7.4).

The Hg^2+^-quenched GQDs as the fluorescent turn-off sensor was studied in detail. Fig. 6 shows the effect of an increasing concentration of Hg^2+^ on the fluorescent intensity of the GQDs. It was found that their fluorescent intensity gradually decreased upon increasing the Hg^2+^ concentration. A linear relationship was obtained in the range of 0–30 μM when the maximum fluorescent intensity at 465 nm was plotted against the Hg^2+^ concentration. At 5 μM of Hg^2+^, the fluorescent intensity of the GQDs was almost quenched with the relative quenching effect of 83.47%. Therefore, this concentration was used for further study.

**Fig. 7:**
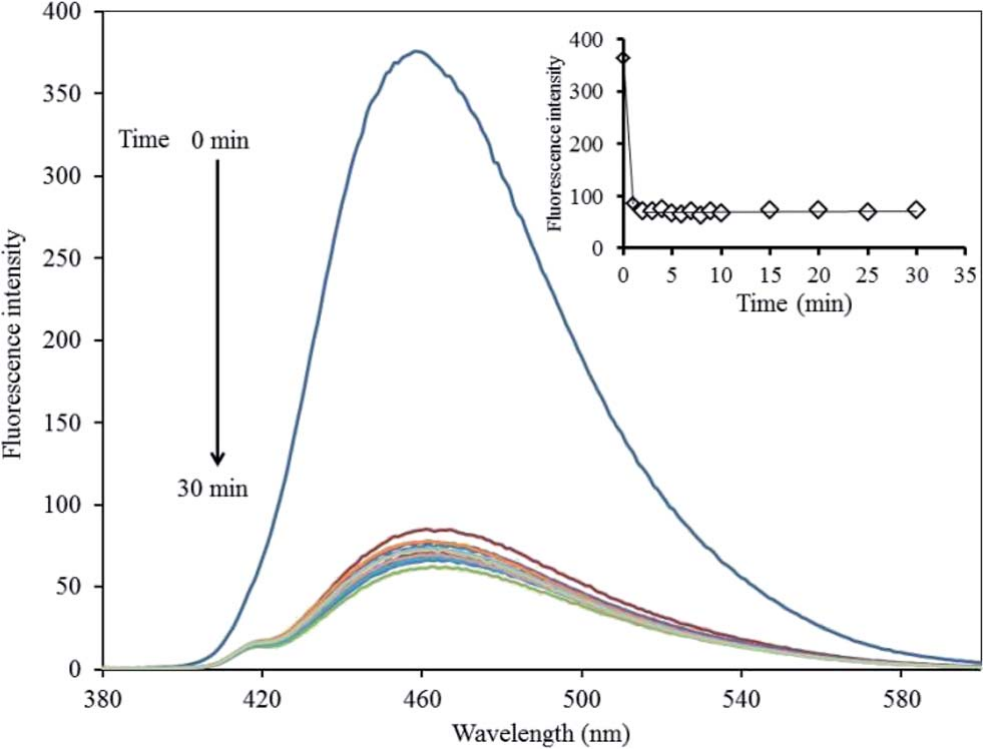
Changes in the emission spectra of the GQDs at different reaction times using 5 μM Hg^2+^ in 0.1 M phosphate buffer (pH 7.4).

To improve the performance of the quenching effect of Hg^2+^, some experimental conditions, including reaction time and solution pH, were optimized. After the addition of 5 μM Hg^2+^, the fluorescent (FL) spectrum of the GQDs solution (20 mg L^−1^ GQDs, pH 7) was recorded within a minute interval. Thus, the FL intensity recorded at 465 nm decreased as much as possible to about 82% within the first minute after the addition of Hg^2+^ (Fig. 7), and then, the FL intensity remained constant over the next 30 min. These results suggested that the quenching process is very rapidly completed in a short time. Besides, the quenching effect of GQDs could only occur if the Hg^2+^ was needed to be detected rapidly.

**Fig. 8:**
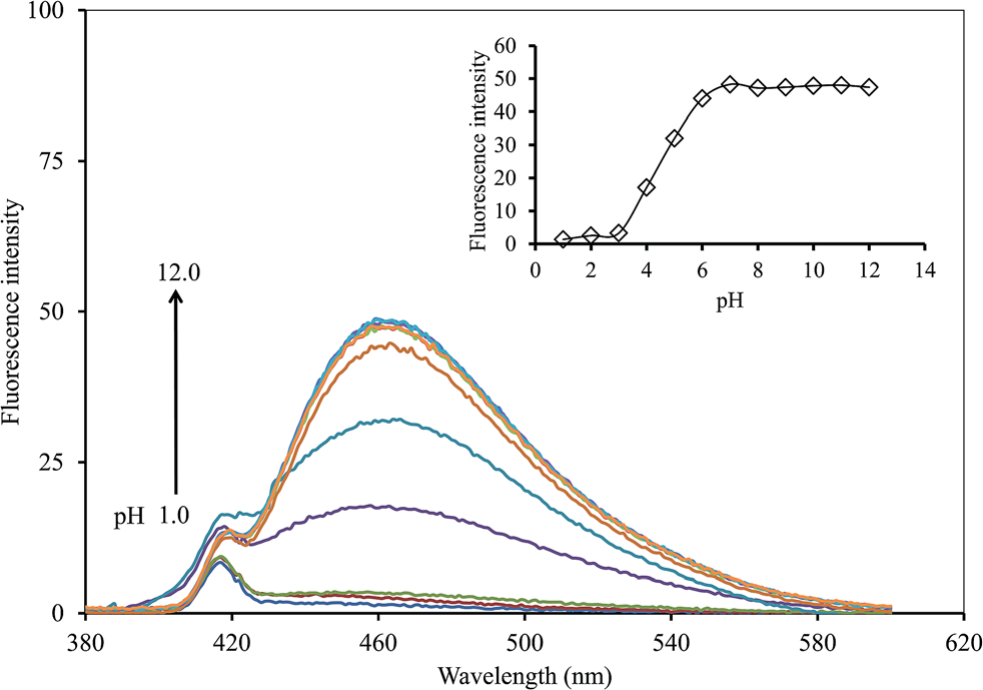
Changes in the emission spectra of the GQDs at different pH values using 5 μM Hg^2+^ and 5 min reaction time.

In terms of the solution pH, it has a strong effect on the FL intensity of the GQDs and thus, it is an important factor for this sensing system. A series of both acetate and phosphate buffer solutions (each 0.1 M) at different pH values were prepared, and the GQDs solution (20 mg L^−1^ final concentration) together with the 5 μM Hg^2+^ were added successively to each of the buffer solutions (pH range 1.0–12.0). The FL intensity of the GQDs at 465 nm was recorded from each of the two buffer solutions, namely with or without added Hg^2+^ (Fig. 8). The FL intensity of the GQDs increased gradually within the pH range 1.0–3.0, then increased sharply in the pH range 3.0–7.0, until finally, it was stabilized under alkaline conditions. This implied that the total quenching effect of the Hg^2+^ would be at pH > 7.0. Thus, the solution pH for highly sensitive and stable Hg^2+^-quenched GQDs was set to pH 7.4.

### Masking effect of CN^−^ and I^−^ for the Hg^2+^–GQDs fluorescent turn-on sensor

**Fig. 9:**
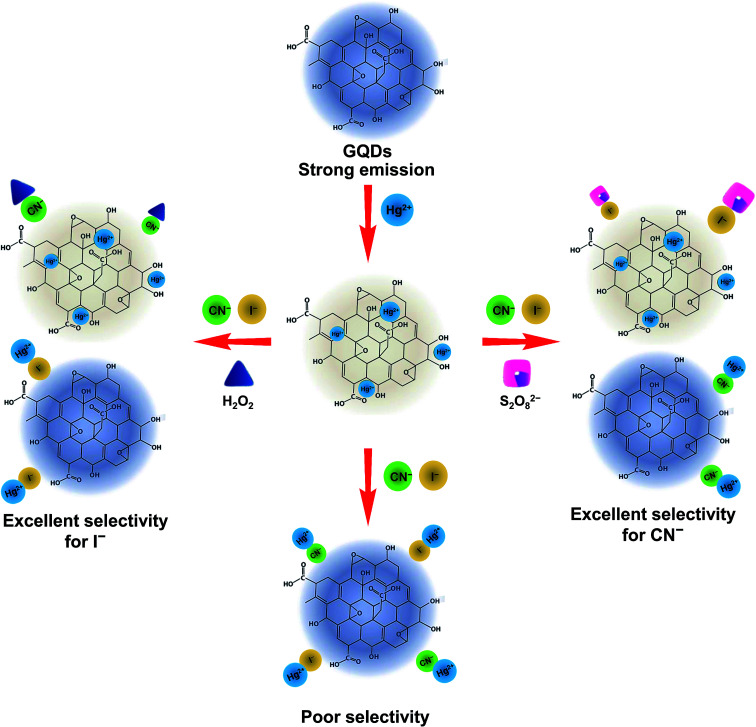
Schematic illustration for turn-off/on fluorescent sensor for the Hg-GQDs-based dual sensing for CN^−^ and I^−^ ions.

**Fig. 10:**
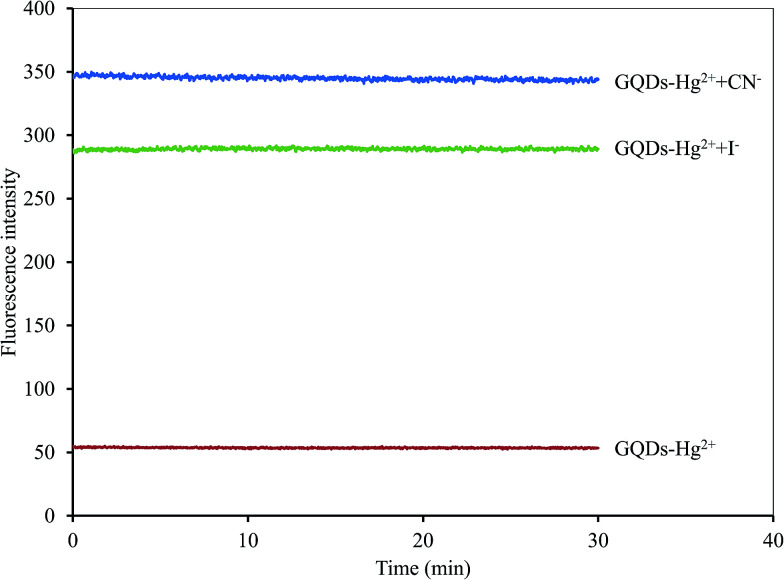
Effect of reaction time on the fluorescent intensity of the Hg^2+^–GQDs by both CN^−^ and I^−^ ions using 5 μM Hg^2+^ and each 10 μM of CN^−^ and I^−^ in 0.1 M phosphate buffer (pH 7.4).

A graphical scheme for the fluorescent turn-on sensor of CN^−^ and I^−^ is shown in Fig. 9. The GQDs show strong blue fluorescence in an aqueous buffer solution. When Hg^2+^ was added, it bound to the GQDs, resulting in a strong quenching effect being observed. Upon the addition of a CN^−^ or I^−^, both CN^−^ and I^−^ react with the Hg^2+^ in the GQDs solution. The Hg^2+^–GQDs complex might be dissociated, and the GQDs fluorescence itself then recovered, which could be used to quantify the CN^−^ or I^−^ under a suitable masking agent of S_2_O_8_^2−^ and H_2_O_2_ for the detection of CN^−^ and I^−^, respectively. Thus, to improve the detection performance of both CN^−^ and I^−^, the experimental conditions, including reaction time, solution pH and concentration of the masking agents, were optimized in detail. After the addition of 10 μM CN^−^ or I^−^, the FL spectrum of the Hg^2+^–GQDs was recorded at a minute time interval. It was found that the FL intensity recorded at 465 nm rapidly increased and kept constant within a minute (Fig. 10), and then still remained constant for a further 30 min.

**Fig. 11:**
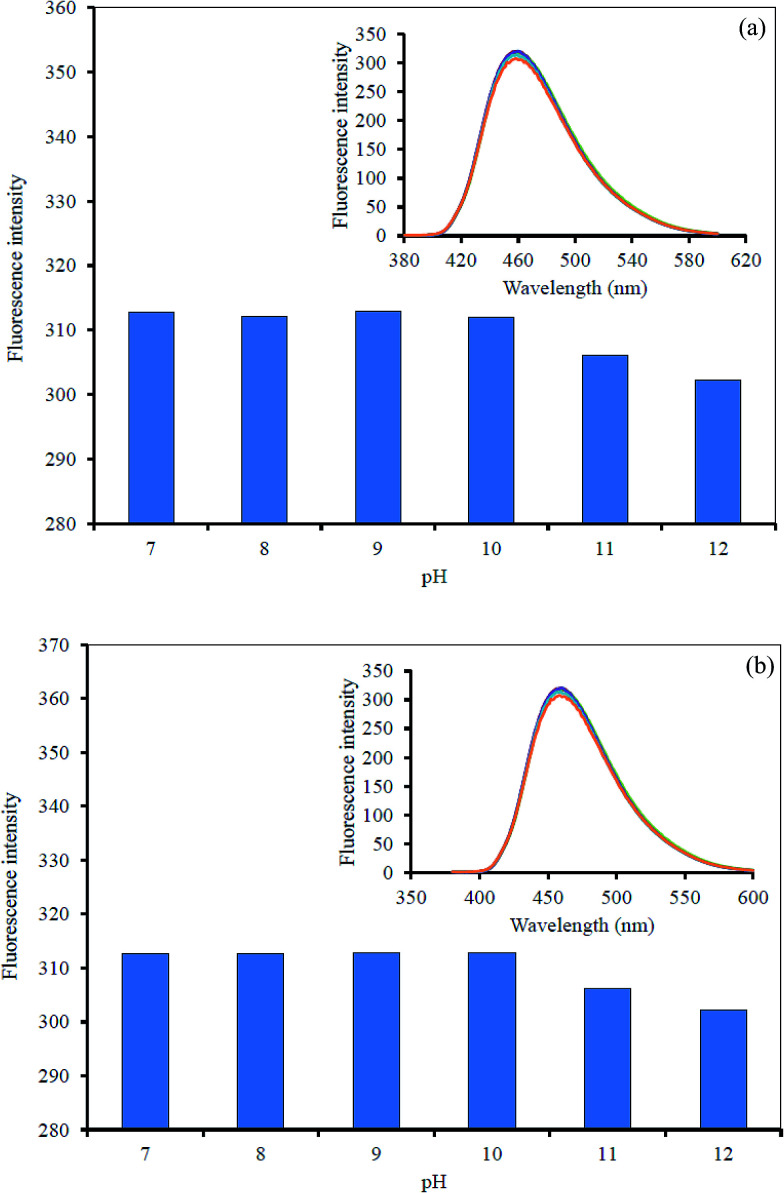
Effect of the solution pH on the fluorescent intensity of the Hg^2+^–GQDs by: (a) CN^−^ and (b) I^−^.

However, the effect of solution pH was also considered for the sensing system. A series of phosphate buffer (0.1 M) at different pH values was used, and the quenched GQDs solution together with 10 μM CN^−^ or I^−^ solution were added successively to each different buffer at pH 7.0–12.0. The FL intensity was recorded from each solution of the Hg^2+^–GQDs + CN^−^ and Hg^2+^–GQDs + I^−^ (Fig. 11). The FL intensity of the quenched GQDs after the addition of either CN^−^ or I^−^ was unchanged in the pH range 7.0–9.0 and then slightly decreased in the pH range 10.0–12.0. It was evident that the fluorescent enhancing effect was highly critical at about pH 9.0.

**Fig. 12:**
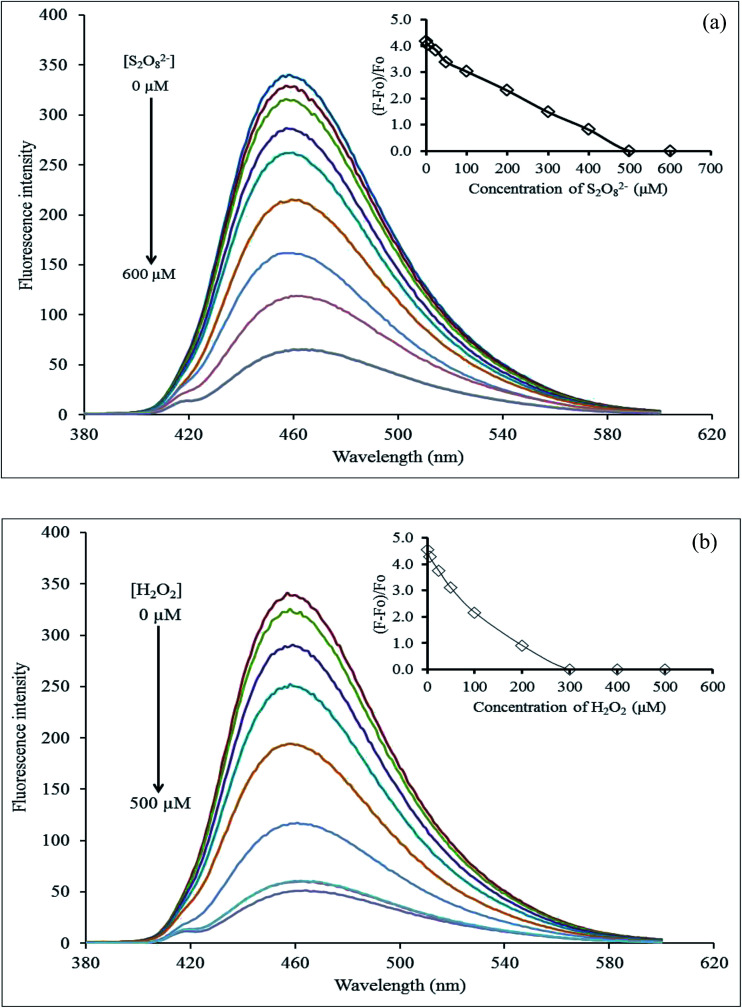
Effect of the masking agent concentration: (a) S_2_O_8_^2−^ and (b) H_2_O_2_ for quenching of the Hg^2+^–GQDs after the addition of I^−^ and CN^−^ ions.

The effect of the masking agent concentration was inevitably investigated. The Hg^2+^–GQDs solution together with 10 μM CN^−^ or I^−^ solution were added successively to each different concentration of either S_2_O_8_^2−^ (0–600 μM) or H_2_O_2_ (0–500 μM). The FL intensity was immediately recorded from each of those solutions (Fig. 12). From the results, it was evident that the FL intensity of Hg^2+^–GQDs decreased with an increasing S_2_O_8_^2−^ in the range 0–500 μM, and then kept constant, while that of the Hg^2+^–GQDs also decreased with the increasing H_2_O_2_ in the range 0–300 μM. Therefore, this implied that the suitable concentrations of the masking agents are 500 and 300 μM for S_2_O_8_^2−^ and H_2_O_2_, respectively.

In aqueous media, the positively charged Hg^2+^ ions tend to be adsorbed on the surface of the negatively charged GQDs because of the electrostatic interaction during the quenching process. Hg^2+^ can quench the FL of GQDs because this facilitates non-radiative electron/hole recombination and annihilation through an effective electron-transfer process,^13^ however, the quenched Hg^2+^–GQDs still possess the potential of emitting FL, and a reversible Hg^2+^ desorption, caused by CN^−^ and I^−^, will lead to “Off–On” FL switching. One explanation for the enhancement process is that CN^−^ and I^−^ can form with the Hg^2+^, which is much stronger than the electrostatic interaction. Thus, the Hg^2+^ cations tend to separate from the GQDs as the amount of CN^−^ and I^−^ increases. Thus, the FL of GQDs will be restored and enhanced linearly within a certain concentration range of CN^−^ and I^−^. Moreover, to enhance the selectivity for each CN^−^ and I^−^ detection, we used H_2_O_2_ and S_2_O_8_^2^ as masking agents for CN^−^ and I^−^, respectively. In fact, H_2_O_2_ possesses a strong oxidation ability for CN^−^ (CN^−^ + H_2_O_2_ → CNO^−^ + H_2_O; *K* = 4.9 × 10^75^) under alkaline pH conditions.^31,62,63^ Therefore, the use of H_2_O_2_ as the masking agent greatly suppresses the interfering matrix from the CN^−^, allowing the Hg^2+^–GQDs sensor to exhibit an excellent selectivity for I^−^. In addition; the oxidation of I^−^ to molecular iodine (I_2_) using Na_2_S_2_O_8_ (S_2_O_8_^2−^ + 2I^−^ → 2SO^2−^ + I_2_; *K* = 7 × 10^15^) can mask the I^−^ ions.^62^

### Selectivity of the Hg^2+^–GQDs fluorescent turn-on sensor for CN^−^ and I^−^ detection

**Fig. 13:**
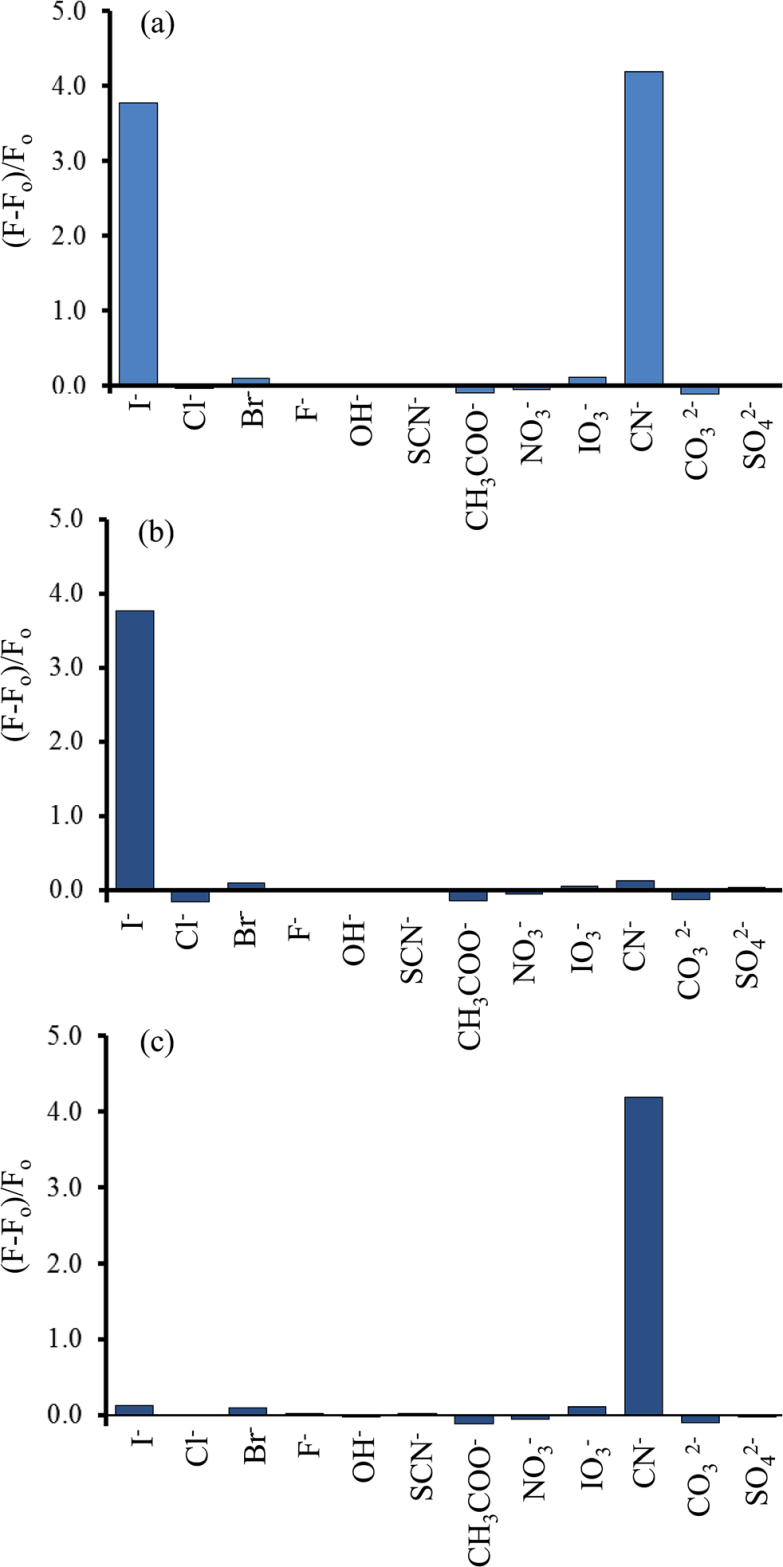
Selectivity of the Hg^2+^–GQDs fluorescent sensor for various anions (10 μM) in the (a) absence of the masking agent used, and in the presence of (b) H_2_O_2_ (300 μM) and (c) S_2_O_8_^2−^ (500 μM).

**Fig. 14:**
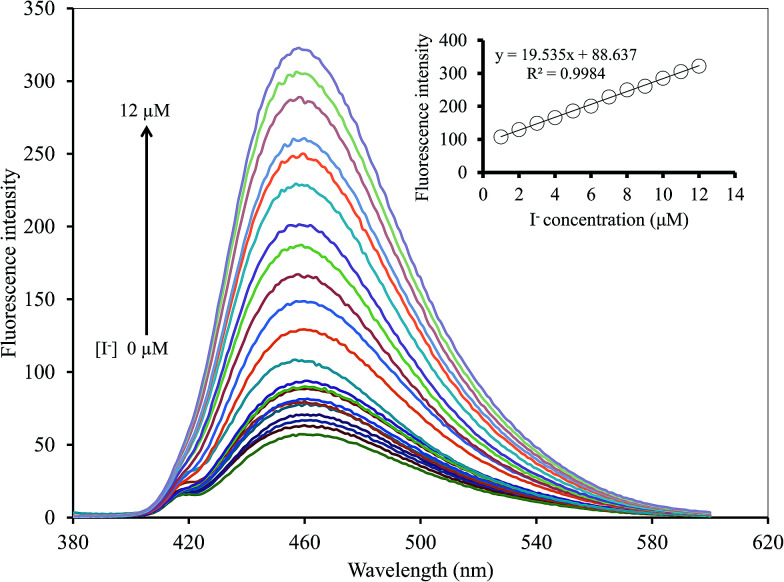
Changing in the emission spectra of the Hg^2+^–GQDs at different I^−^ concentrations.

To study the selectivity of the Hg^2+^–GQDs sensor for CN^−^ and I^−^ detection, the intensity ratios of (*F* − *F*_0_)/*F*_0_ for the Hg^2+^–GQDs in the presence of various anions, including iodide (I^−^), chloride (Cl^−^), bromide (Br^−^), fluoride (F^−^), hydroxide (OH^−^), thiocyanate (SCN^−^), acetate (CH_3_COO^−^), nitrate (NO_3_^−^), iodate (IO_3_^−^), cyanate (CN^−^), carbonate (CO_3_^2−^) and sulfate (SO_4_^2−^), were obtained. The concentration of each anion was 10 μM, same as that of the CN^−^ or I^−^ in the assay solution. Fig. 13a shows that the addition of CN^−^ and I^−^ to those solutions resulted in an apparent recovery of the fluorescent intensity (turn-on), whereas the other remaining anions had no effect under the same experimental conditions. Using H_2_O_2_ (300 mM) as the masking agent greatly suppressed the interfering matrix from the CN^−^, allowing the Hg^2+^–GQDs sensor to exhibit an excellent selectivity for I^−^ (Fig. 13b). The Hg^2+^–GQDs in 0.1 M phosphate buffer solution (pH 9.0) containing 300 mM H_2_O_2_ exhibited a selectivity of about 300-fold for I^−^ over the other anions. Under the optimal conditions, this could be used to determine the trace level of I^−^ by monitoring the fluorescence enhancement [(*F* − *F*_0_)/*F*_0_] of the Hg^2+^–GQDs solution containing H_2_O_2_. When the iodide concentration was increased, a gradual increase in the fluorescent intensity of the probe solution was observed (Fig. 14). The plots of relative fluorescence [(*F* − *F*_o_)/*F*_o_] exhibited a good linearity over the I^−^ concentration ranging from 1 to 12 μM with a correlation coefficient (*R*^2^) of 0.9984. This proposed method enabled the selective sensing of I^−^ with a limit of detection (LOD) of 0.2 μM with an S/N ratio of 3.

**Fig. 15:**
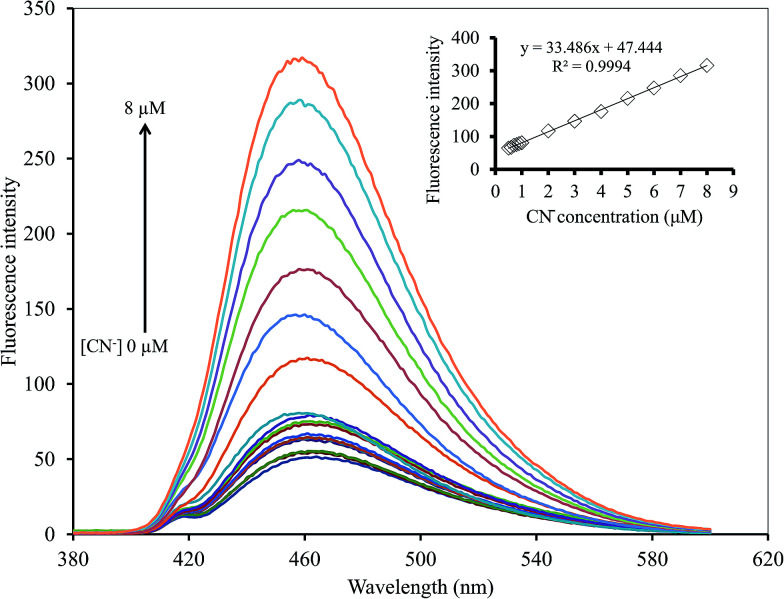
Changes in the emission spectra of the Hg^2+^–GQDs at different CN^−^ concentrations.

In addition, the effect of S_2_O_8_^2−^ on the Hg^2+^–GQDs system was also studied. For this purpose, the fluorescent sensor was an extensive choice for CN^−^ with respect to the other anion species in the presence of 500 mM S_2_O_8_^2−^ between the relative fluorescent intensity and the concentration in the range 0–8 μM (*R*^2^ = 0.9994) (Fig. 15). The LOD of CN^−^ was 0.17 μM with an S/N ratio of 3. The results suggested that the Hg^2+^–GQDs fluorescent turn-on sensor was very sensitive for monitoring both CN^−^ and I^−^ at trace levels in real samples.

## Method validation

### Performance evaluation

The analytical characteristics of the proposed method were validated under the optimized conditions in terms of linearity, limit of detection, limit of quantification and precision (expressed as the relative standard deviation, RSD, of the calibration slope obtained from both intra-day and inter-day analysis) to estimate the efficiency and feasibility of the method for use with drinking water samples. The results obtained are shown in Table 1. The linearity ranges were found to be 0.5–8.0 μM (*R*^2^ = 0.9994) and 1.0–12.0 μM (*R*^2^ = 0.9984) for CN^−^ and I^−^, respectively. Their linear calibration graphs were as follows: *y* = 33.486*x* + 47.444 and *y* = 19.535*x* + 88.637 (where *y* is the fluorescence intensity and *x* is the concentration of CN^−^ or I^−^). The limits of detection (LOD) defined as 3SD/*m* (where SD is the standard deviation of a very low concentration of CN^−^ or I^−^ and *m* is the slope of the calibration graph) were 0.17 and 0.20 μM for CN^−^ and I^−^, respectively. While the limits of quantification (LOQ), defined as 10SD/*m*, were 0.99 and 0.78 μM for CN^−^ and I^−^, respectively. The precision, expressed as % RSD of the slope of the calibration graph, was evaluated in terms of repeatability (*n* = 9, intra-day RSD) and was 2.33% and 1.03%, while reproducibility (work performed during 11 consecutive days, inter-day RSD) was 4.92% and 3.05% for CN^−^ and I^−^, respectively.

**Table 1:** Analytical characteristics of GQDs–Hg system for the determination of CN^−^ and I^−^ ions

Analytical parameter	Analytical feature	
	CN^−^	I^−^
Linear range (μM)	0.5–8.0	1.0–12.0
Linear equation	*y* = 33.486*x* + 47.444	*y* = 19.535*x* + 88.637
Correlation coefficient (*R*^2^)	0.9994	0.9984
Limit of detection (μM, *n* = 11)	0.17	0.20
Limit of quantification (μM, *n* = 11)	0.99	0.78
Relative standard deviation (%) for: intra-day analysis (*n* = 9)	2.33	1.03
Relative standard deviation (%) for: inter-day analysis (*n* = 11)	4.92	3.05

### Analysis of real samples

To demonstrate the applicability and reliability of the proposed method, it was successfully applied to 15 samples of drinking water, including five brands of water (brand 1–5). The contents of CN^−^ and I^−^ in these samples were obtained as shown in Table 2. The results showed that both CN^−^ and I^−^ were not found in all the samples studied. In addition, to evaluate the matrix effect the accuracy of the method was verified by calculating the recovery study in the real samples. Each sample was spiked with three concentrations (0.5, 3.0 and 7.5 μM) of the standard solution of both CN^−^ and I^−^. Then, the relative percentage recoveries were calculated as follows:^64^% recovery = [(*C*_found_ − *C*_real_)/*C*_added_] × 100where, *C*_found_, *C*_real_ and *C*_added_ are the concentration of analyte after the addition of the known amount of standard in the real sample, the concentration of analyte in the real sample and the concentration of the known amount of standard that was spiked in the real sample, respectively. From the results (Table 2), it was found that the recoveries of the proposed method expressed as the mean percentage (*n* = 3) were in the range of 72.97–112.41 and 78.61–117.09% for CN^−^ and I^−^, respectively. This demonstrates that this method provides acceptable recovery for the determination of both CN^−^ and I^−^ in these real samples. Therefore, it was concluded that the matrix effect in these samples was negligible for all on the performance of the proposed method.

**Table 2:** The contents and their recoveries of CN^−^ and I^−^ in drinking water samples using the GQDs–Hg system (*n* = 3)

Drinking water sample	CN^−^			I^−^		
	Added (μM)	Found (μM)	Recovery (%) ± SD	Added (μM)	Found (μM)	Recovery (%) ± SD
Brand 1	0.0	0.00	—	0.0	0.00	—
	0.5	0.36	77.40 ± 4.98	0.5	0.44	88.99 ± 3.92
	3.0	3.30	110.86 ± 8.27	3.0	2.77	92.31 ± 5.74
	7.5	7.76	103.81 ± 5.44	7.5	7.35	98.02 ± 4.69
Brand 2	0.0	0.00	—	0.0	0.00	—
	0.5	0.36	72.97 ± 6.11	0.5	0.34	78.61 ± 2.97
	3.0	2.93	97.70 ± 3.67	3.0	2.72	90.53 ± 5.48
	7.5	7.00	93.30 ± 2.79	7.5	7.26	96.75 ± 3.61
Brand 3	0.0	0.00	—	0.0	0.00	—
	0.5	0.56	112.41 ± 7.33	0.5	0.59	117.09 ± 7.08
	3.0	3.02	100.71 ± 5.23	3.0	2.90	96.72 ± 5.63
	7.5	7.37	98.20 ± 4.71	7.5	7.46	99.47 ± 4.75
Brand 4	0.0	0.00	—	0.0	0.00	—
	0.5	0.41	81.52 ± 6.51	0.5	0.43	86.69 ± 3.44
	3.0	3.21	106.94 ± 4.15	3.0	3.07	102.29 ± 4.74
	7.5	7.69	102.53 ± 5.20	7.5	7.42	98.94 ± 5.31
Brand 5	0.0	0.00	—	0.0	0.00	—
	0.5	0.41	81.52 ± 3.73	0.5	0.51	102.48 ± 4.22
	3.0	3.14	104.61 ± 7.00	3.0	2.87	95.70 ± 5.09
	7.5	7.41	98.80 ± 6.30	7.5	7.32	97.66 ± 3.17

## Conclusions

The proposed method described was a highly sensitive and selective turn-on fluorescent sensor for cyanide and iodide in water samples using Hg^2+^–GQDs with the featured marking agents as a novel nanosensor. The quenched GQDs were almost stable in alkaline conditions. The prominent advantage of the method is its simplicity and rapidity. In the presence of the masking agent, persulfate permitted the detection of CN^−^ with selectivity. It is also demonstrated that in the presence of hydrogen peroxide, the Hg^2+^–GQDs selectively could detect I^−^. Under the optimum conditions, their analytical features of merit were validated. Some common interfering ions were shown not to affect the determination of CN^−^ and I^−^. The proposed method was applied for the determination of both CN^−^ and I^−^ in real samples successfully with the acceptable recovery ranges of 72.97–112.41% and 78.61–117.09% for CN^−^ and I^−^, respectively.

## Conflicts of interest

The authors have declared no conflict of interest.

## Supplementary Material

## References

[cit1] Huang S., Qiu H., Zhu F., Lu S., Xiao Q. (2015). Microchim. Acta.

[cit2] Zhang Q., Song C., Zhao T., Fu H. W., Wang H. Z., Wang Y. J., Kong D. M. (2015). Biosens. Bioelectron..

[cit3] Myung S., Solanki A., Kim C., Park J., Kim K. S., Lee K. B. (2011). Adv. Mater..

[cit4] Razmi H., Mohammad-Rezaei R. (2013). Biosens. Bioelectron..

[cit5] Nahain A. A., Lee J. E., In I., Lee H., Lee K. D., Jeong J. H., Park S. Y. (2013). Mol. Pharmaceutics.

[cit6] Kumar G. S., Roy R., Sen D., Ghorai U. K., Thapa R., Mazumder N., Saha S., Chattopadhyay K. K. (2014). Nanoscale.

[cit7] Yuan X., Liu Z., Guo Z., Ji Y., Jin M., Wang X. (2014). Nanoscale Res. Lett..

[cit8] Valizadeh H., Shomali A., Nourshargh S., Mohammad-Rezaei R. (2015). Dyes Pigm..

[cit9] Lai S. K., Luk C. M., Tang L., Teng K. S., Lau S. P. (2015). Nanoscale.

[cit10] Ting S. L., Ee S. J., Ananthanarayanan A., Leong K. C., Chen P. (2015). Electrochim. Acta.

[cit11] Song Y., Chen S. (2014). ACS Appl. Mater. Interfaces.

[cit12] Chen S., Hai X., Chen X. W., Wang J. H. (2014). Anal. Chem..

[cit13] Li Z., Wang Y., Ni Y., Kokot S. (2015). Sens. Actuators, B.

[cit14] Wang F., Gu Z., Lei W., Wang W., Xia X., Hao Q. (2014). Sens. Actuators, B.

[cit15] Tam T. V., Trung N. B., Kim H. R., Chung J. S., Choi W. M. (2014). Sens. Actuators, B.

[cit16] Zhou X., Ma P., Wang A., Yu C., Qian T., Wu S., Shen J. (2015). Biosens. Bioelectron..

[cit17] Tian J., Zhao H., Quan X., Zhang Y., Yu H., Chen S. (2014). Sens. Actuators, B.

[cit18] Qian Z. S., Shan X. Y., Chai L. J., Ma J. J., Chen J. R., Feng H. (2014). Biosens. Bioelectron..

[cit19] Wang X., Sun X., Lao J., He H., Cheng T., Wang M., Wang S., Huang F. (2014). Colloids Surf., B.

[cit20] Wu Z., Li W., Chen J., Yu C. (2014). Talanta.

[cit21] Sun R., Wang Y., Ni Y., Kokot S. (2014). Talanta.

[cit22] Li Z., Wang Y., Ni Y., Kokot S. (2015). Spectrochim. Acta, Part A.

[cit23] Amjadi M., Manzoori J. L., Hallaj T. (2014). J. Lumin..

[cit24] Liu S., Kang J., Cao X., Yue X. (2016). Spectrochim. Acta, Part A.

[cit25] Udhayakumari D., Velmathi S., Boobalan M. S. (2015). J. Fluorine Chem..

[cit26] Sharma D., Ashok Kumar S. K., Sahoo S. K. (2014). Tetrahedron Lett..

[cit27] Su X., Guo L., Ma Y., Li X. (2016). Spectrochim. Acta, Part A.

[cit28] Bothra S., Kumar R., Pati R. K., Kuwar A., Choi H. J., Sahoo S. K. (2015). Spectrochim. Acta, Part A.

[cit29] Zeng J., Cao Y., Lu C. H., Wang X., Wang Q., Wen C. Y., Qu J. B., Yuan C., Yan Z. F., Chen X. (2015). Anal. Chim. Acta.

[cit30] Pati P. B. (2016). Sens. Actuators, B.

[cit31] Wei S. C., Hsu P. H., Lee Y. F., Lin Y. W., Huang C. C. (2012). ACS Appl. Mater. Interfaces.

[cit32] Cheng J., Jandik P., Avdalovic N. (2005). Anal. Chim. Acta.

[cit33] Minakata K., Yamagishi I., Kanno S., Nozawa H., Suzuki M., Suzuki O. (2010). J. Chromatogr. B: Anal. Technol. Biomed. Life Sci..

[cit34] Mazumder A., Kumar A., Dubey D. K. (2013). J. Chromatogr. A.

[cit35] Bermejo-Barrera P., Fernᾴandez-Sanchez L. M., Aboal-Somoza M., Anllo-Sendın R. M., Bermejo-Barrera A. (2001). Microchem. J..

[cit36] Yan F., Reddy C. V. G., Zhanga Y., Vo-Dinh T. (2010). Ecotoxicol. Environ. Saf..

[cit37] Limchoowong N., Sricharoen P., Techawongstien S., Chanthai S. (2016). Food Chem..

[cit38] Chen S. H., Wu H. L., Tanaka M., Shono T., Funazo K. (1990). J. Chromatogr. A.

[cit39] Minakata K., Yamagishi I., Kanno S., Nozawa H., Suzuki M., Suzuki O. (2010). J. Chromatogr. B: Anal. Technol. Biomed. Life Sci..

[cit40] Noroozifar M., Khorasani-Motlagh M., Hosseini S. N. (2005). Anal. Chim. Acta.

[cit41] Limchoowong N., Sricharoen P., Techawongstien S., Kongsri S., Chanthai S. (2017). J. Braz. J. Braz. Chem. Soc..

[cit42] Safavi A., Maleki N., Shahbaazi H. R. (2004). Anal. Chim. Acta.

[cit43] El-Shishtawy R. M., Al-Zahrani F. A. M., Al-amshany Z. M., Asiri A. M. (2017). Sens. Actuators, B.

[cit44] Sukato R., Sangpetch N., Palaga T., Jantra S., Vchirawongkwin V., Jongwohan C., Sukwattanasinitt M., Wacharasindhu S. (2016). J. Hazard. Mater..

[cit45] Li J., Qi X., Wei W., Zuo G., Dong W. (2016). Sens. Actuators, B.

[cit46] Sun Y., Li Y., Ma X., Duan L. (2016). Sens. Actuators, B.

[cit47] Guan R., Chen H., Cao F., Cao D., Deng Y. (2013). Inorg. Chem. Commun..

[cit48] Fu L., Li C., Li Y., Chen S., Long Y., Zeng R. (2017). Sens. Actuators, B.

[cit49] Xiao Y., Zhang Y., Huang H., Zhang Y., Du B., Chen F., Zheng Q., He X., Wang K. (2015). Talanta.

[cit50] Nabavi S., Alizadeh N. (2014). Sens. Actuators, B.

[cit51] Novoselov K. S., Geim A. K., Morozov S. V., Jiang D., Zhang Y., Dubono S. V. (2004). Science.

[cit52] Xie M., Su Y., Lu X., Zhang Y., Yang Z., Zhang Y. (2013). Mater. Lett..

[cit53] Li Y., Hu Y., Zhao Y., Shi G., Deng L., Hou Y. (2011). Adv. Mater..

[cit54] Lu J., Yeo P. S. E., Gan C. K., Wu P., Loh K. P. (2011). Nat. Nanotechnol..

[cit55] Dong Y., Shao J., Chen C., Li H., Wang R., Chi Y., Lin X., Chen G. (2012). Carbon.

[cit56] Li L., Wu G., Yang G., Peng J., Zhao J., Zhu J. J. (2013). Nanoscale.

[cit57] Cushing S. K., Li M., Huang F., Wu N. (2014). ACS Nano.

[cit58] Castillo N., Luna E., Arellano M. G., Ocampo P. C., Flores S. O., Conde A. (2013). Journal of Multifunctional Materials & Photoscience.

[cit59] Wang H., Hao Q., Yang X., Lu L., Wang X. (2009). Electrochem. Commun..

[cit60] Chai F., Wang T., Li L., Liu H., Zhang L., Su Z., Wang C. (2010). Nanoscale Res. Lett..

[cit61] Lu W. B., Qin X. Y., Liu S., Chang G. H., Zhang Y. W., Luo Y. L., Asiri A. M., Al-Youbi A. O., Sun X. P. (2012). Anal. Chem..

[cit62] Secco F., Celsi S. (1971). J. Chem. Soc. A.

[cit63] Yeddou A. R., Chergui S., Chergui A., Halet F., Hamza A., Nadjemi B., Ould-Dris A., Belkouch J. (2011). Miner. Eng..

[cit64] Limchoowong N., Sricharoen P., Techawongstien S., Chanthai S. (2017). Food Chem..

